# The Effect of Hops (*Humulus lupulus* L.) Extract Supplementation on Weight Gain, Adiposity and Intestinal Function in Ovariectomized Mice

**DOI:** 10.3390/nu11123004

**Published:** 2019-12-07

**Authors:** Alison K. Hamm, Daniel K. Manter, Jay S. Kirkwood, Lisa M. Wolfe, Kimberly Cox-York, Tiffany L. Weir

**Affiliations:** 1 USDA ARS Soil-Plant-Nutrient Research Unit, Fort Collins, CO 80526, USA; alison.hamm@usda.gov (A.K.H.); daniel.manter@usda.gov (D.K.M.); 2 Metabolomics Core Facility, University of California, Riverside, CA 92521, USA; jkirk@ucr.edu; 3 Proteomics and Metabolomics Facility, Colorado State University, Fort Collins, CO 80523, USA; lisa.wolfe@colostate.edu; 4 Department of Food Science and Human Nutrition, Colorado State University, Fort Collins, CO 80523, USA

**Keywords:** adiposity, dysbiosis, hops, menopause, microbiota, 8-prenylnaringenin, obesity, ovariectomy

## Abstract

Estrogen decline during menopause is associated with altered metabolism, weight gain and increased risk of cardiometabolic diseases. The gut microbiota also plays a role in the development of cardiometabolic dysfunction and is also subject to changes associated with age-related hormone changes. Phytoestrogens are plant-based estrogen mimics that have gained popularity as dietary supplements for the treatment or prevention of menopause-related symptoms. These compounds have the potential to both modulate and be metabolized by the gut microbiota. Hops (*Humulus lupulus* L.) contain potent phytoestrogen precursors, which rely on microbial biotransformation in the gut to estrogenic forms. We supplemented ovariectomized (OVX) or sham-operated (SHAM) C57BL/6 mice, with oral estradiol (E2), a flavonoid-rich extract from hops, or a placebo carrier oil, to observe effects on adiposity, inflammation, and gut bacteria composition. Hops extract (HE) and E2 protected against increased visceral adiposity and liver triglyceride accumulation in OVX animals. Surprisingly, we found no evidence of OVX having a significant impact on the overall gut bacterial community structure. We did find differences in the abundance of *Akkermansia muciniphila,* which was lower with HE treatment in the SHAM group relative to OVX E2 treatment and to placebo in the SHAM group.

## 1. Introduction

The onset of the menopause is marked by declining ovarian estrogen production and is associated with long-term health consequences in women. The loss of endogenous estrogen (17-β estradiol; E2) is associated with increased visceral adiposity and risk of metabolic disease, such as cardiovascular disease (CVD), and fatty liver [1,2,3]. Accumulating evidence suggests that estrogen may be an important regulator of the intestinal microbiota [4,5]. Recent research in murine models has shown that loss of endogenous estrogen production through removal of the ovaries by ovariectomy (OVX) alters the composition of intestinal bacteria [6,7,8,9,10]. Gut bacteria are capable of metabolizing numerous exogenous and endogenous compounds, including estrogens, implicating the gut ecosystem in the disease risk associated with menopause. The ‘estrobolome’ is a term describing “the aggregate of enteric bacterial genes whose products are capable of metabolizing estrogens” [11]. The actions of the estrobolome can increase or decrease the bioavailability of endogenous and exogenous estrogens, thus influencing a woman’s lifetime estrogen exposure. This reciprocal interaction between the gut microbiota and estrogens may have important implications in women’s health and determining the risk of developing age-associated diseases.

Aside from the implications for chronic disease risk, menopause is associated with numerous acute physical symptoms. Historically, the first-line treatment was hormone therapy (HT), however the health consequences of HT came under fire with the Women’s Health Initiative (WHI) Study [12] and the demand for HT has declined in the past decade due to its association with increased breast cancer and cardiovascular disease risk [13,14,15]. Subsequent analysis of these studies demonstrated important nuances related to the time and duration of HT [16], but consumers remain cautious and often opt for alternative therapies to address menopausal symptoms and help maintain the beneficial effects of estrogen on health. One potential alternative is the use of botanical supplements that contain estrogen-like compounds, called phytoestrogens. Through the activation of estrogen receptors, phytoestrogens can alleviate uncomfortable menopausal symptoms and may interact with the gut microbiota to mitigate the risk of chronic diseases [17]. Estrogenic compounds have been reported in varying concentrations in soybean, flax and sesame seeds, kudzu root and wild yam. However, flavonoid compounds from hops, *Humulus lupulus*, are among the most potent phytoestrogens identified to date [18,19]. Several studies in murine models and human trials have shown the effectiveness of hop flavonoids in reducing menopausal symptoms while lowering the risk of cardiometabolic diseases through direct flavonoid interaction with endogenous antioxidant and anti-inflammatory pathways [20,21,22,23]. Commercial, hops-based dietary supplements contain a mixture of the flavonoids xanthohumol (XN), isoxanthohumol (IX) and 8-prenylnaringenin (8PN). The estrogenic activity of hop flavonoids is dependent on the gut bacterial metabolism of XN and IX and the phytoestrogen 8PN [24]. Human gut bacteria, including *Eubacterium limosum,* [25] and *Eubacterium ramulus* [26], are able to convert IX to 8PN, providing evidence for a mechanism by which the gut microbiota can influence resultant 8PN exposure. 

We have previously demonstrated that ovariectomized (OVX) rats had a dysbiotic gut microbiota compared to sham-operated controls, characterized by increased Bacteroidetes and higher microbial diversity, driven largely by increases in LPS-associated Gram-negative bacteria [6]. Building on this data, the objectives of the current study were to determine whether similar alterations to the microbiota would occur in a more genetically tractable mouse model of OVX and establish whether estrogen or phytoestrogen treatment could mitigate these changes. We hypothesized that OVX would be associated with increased adiposity and gut dysbiosis, characterized by alteration to the microbiome and increased intestinal permeability and inflammation. We further hypothesized that the replacement of estrogen activity using E2 or phytoestrogen-rich HE would mitigate these effects. Ovariectomy (OVX) or sham (SHAM) surgeries were conducted in 7-month old retired breeder C57BL/6 mice. All animals were fed a purified, phytoestrogen-free diet and randomized to treatment groups, then given either a commercial supplement made from HE, E2, or a placebo carrier oil. Microbiota was measured using 16S DNA sequencing, and functional aspects of the microbiota were determined by quantifying short chain fatty acids and secondary bile acids in fecal material. In vivo intestinal permeability was assessed and inflammation in the intestinal tissue was assayed by a multiplex Luminex-based cytokine analysis. 

## 2. Materials and Methods

### 2.1. Animal Study

**Figure 1:**
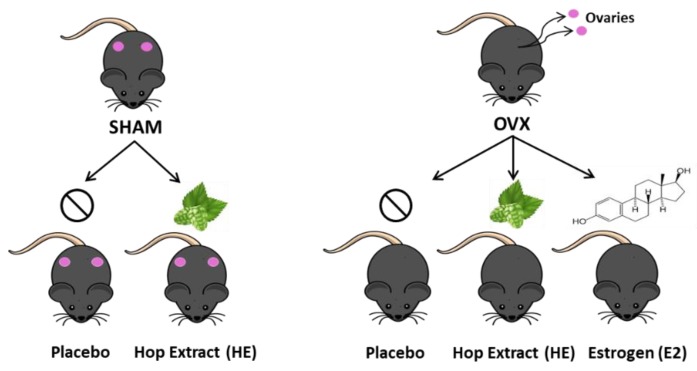
Treatment and control groups used in the study.

Animal conditions met the standards of the Animal Welfare Act regulations and Guide for the Care and Use of Laboratory Animals, and animal care, and procedures were approved by the Colorado State University Institutional Animal Care and Use Committee. Female C57BL/6 7-month old retired breeder mice were obtained from Charles River Laboratories (Wilmington, MA, USA). Retired breeders were chosen to more closely mimic the menopausal period of the mouse life cycle. Upon arrival, mice were housed individually in an environment controlled for temperature, humidity, and light cycle (12 h light:dark). Mice were provided a phytoestrogen-free, standardized, purified, low-fat diet (TD.08113 Harlan, Madison WI, USA) and water ad libitum. After two weeks of acclimation, mice were individually housed and randomized into groups based on average body weight. Under isoflurane anesthesia, mice underwent dorsal entry ovariectomy, conducted by making an incision through the skin and muscle just caudal to the last rib and about 1 cm ventral to the dorsal spinous process of the third lumbar vertebra, followed by ligation and removal of ovaries. Control groups underwent sham surgery, which included exposure without ligation and removal of the ovaries. The muscle was sutured and the skin incision was closed with wound clips. Mice received analgesic (Meloxicam; 1.2 mg/kg) prior to surgery and for 24 h post-surgery. Body weight and food intake were measured weekly for 12 weeks. The five study groups included: OVX Placebo (OVX; *n* = 11), OVX plus hop extract (OVX HE; *n* = 11), OVX plus 17 β-estradiol (OVX E2; *n* = 9), Sham Placebo (SHAM; *n* = 10), and Sham HE (SHAM HE; *n* = 8) (Figure 1).

All mice were maintained on a purified phytoestrogen-free diet (Harlan TD.08113) with 13.8% calories from protein, 76.0% from carbohydrates, and 10.2% from fat. Four to seven days post-surgery, mice began receiving either supplemental 17 β-estradiol (E2; Sigma-Aldrich, St. Louis, MO, USA), hop extract (HE; MetaGenics, Aliso Viejo, CA, USA) or placebo (sesame seed oil). The HE and E2 were suspended in 20 μL sesame oil and dissolved onto 0.2 g of a hazelnut wafer cookie (Quadratini, Loacker^®^), while placebo groups received only the cookie and sesame oil. All animals were provided cookies daily and they were fully consumed within 10 min of administration. Based on previous studies, we administered 56 mg/kg E2 [27] and 400 mg/kg HE [23,28] to the mice daily. This amount of HE was composed of 5.1 g/mg 8-prenylnaringenin (8PN), and 6.3 g/mg xanthohumol (XN), as determined by UHPLC-MS of the powdered extract. Considering the diet plus cookie, mice obtained 15.6% of their total calories from fat, 12.7% from protein and 71.9% from carbohydrate (CHO) (Table 1).

### 2.2. Tissue Collection

Mice were fasted for 4 h before termination, anesthetized with carbon dioxide and euthanized by exsanguination. Blood was collected and allowed to clot at room temperature for 30 min, then was incubated on ice for 90 min before centrifugation at 8000 rpm for 10 min at 4 °C. Serum was drawn off and stored at ‒80 °C until further analysis. The liver, ileum, proximal colon and distal colon were excised, cleaned with saline and immediately frozen in liquid nitrogen. Adipose tissue, uterus and cecum tissues were weighed and recorded prior to freezing. Ovariectomy was confirmed by removing, weighing, and visually inspecting the uterus and confirming the absence of ovaries. 

### 2.3. Liver Triglycerides and Liver Gene Expression

Loss of estrogen is often associated with increased central adiposity and ectopic lipid accumulation, with the liver often being one of the first tissues affected. Thus, we measured liver triglycerides to establish the effects of OVX and the various supplement treatments on lipid accumulation in the liver. Liver tissue was digested in ethanolic potassium hydroxide, purified by two ethanol purification steps and precipitated with magnesium chloride. The supernatant was assayed using the Cayman (Ann Arbor, MI, USA) triglyceride colorimetric kit per manufacturer’s instructions. Samples were measured against a standard curve (0–200 mg/dL), and measurements were normalized to liver weight. To further examine how OVX and the supplementation with E2 or HE influenced lipid metabolism in the liver, we examined the expression of several genes involved in de novo lipogenesis and lipid transport. Liver RNA was isolated with TRIzol reagent (Life Technology, Grand Island, NY, USA) based on the manufacturer’s instructions and quantified using a Q3000 UV spectrophotometer (Quawell Technology Inc, San Jose, CA, USA). Isolated RNA (425.8–1546.9 ng/mL) was synthesized into cDNA using iScript kit (Bio-Rad, Hercules, CA, USA). Samples were run in duplicate, and the expression of ATP-binding cassette subfamily G (ABCG5 and ABCG8), steroid regulatory binding protein 1c (SREBP1c), fatty acid synthase (FASn), Hormone sensitive lipase (LIPE), and acetyl-CoA carboxylase (AcCoA) were compared among treatment groups. Primer sequences are listed in Table 2. Quantitative PCR was performed with BioRad SoAdvanced™ SYBR Green Supermix on CFX96™ thermal cycler (Bio-Rad, Hercules, CA, USA). Thermal cycling conditions were as follows: 3 min 95 °C, 40 cycles of 95 °C for 10 s, 58 °C for 30 s, and 72 °C for 30 s, followed by 72 °C for 3 min. Results were normalized to the reference gene beta-2-microglobulin (β2M) and reported as relative expression change from cycle threshold.

### 2.4. Quantification of Hop Prenylflavonoids in Hop Extract and Serum

To confirm levels of specific prenylflavonoids in theHE powder, we used metabolomic analysis to quantify XN and 8PN. Eight milligrams of powder was diluted with 500 µL of cold MeOH containing the internal standards 2,4-dihydroxychalcone and naringenin at 100 ng/mL. A further 1000-fold dilution was required to prevent signal saturation. To quantify levels of XN, IX and 8PN in the blood after oral administration, we used four mice from the same cohort as the study animals (identical to study animals in age, source, and environmental conditions). Mice were fasted for 6 h, and HE was suspended in sesame oil at the same mg/kg administered daily to study mice (400 mg/kg). The treatment was dropped onto the back fur and the mice licked it off within 5 min. Serum samples were collected over the next 30 h from tail vein blood. To quantify levels of HE flavonoids and their metabolites in our study mice, serum was collected from OVX HE and SHAM HE mice at termination. Mice were fasted for 4 h and had their last cookie with HE treatment 20 h prior to serum collection. Mouse serum samples were extracted using a protein precipitation protocol. To a 1.5 mL eppendorf tube, 19 µL of serum was added, followed by 80 µL of cold MeOH containing the internal standards 2,4-dihydroxychalcone and naringenin at 100 ng/mL. After vortexing for 30 s, samples were centrifuged 10 min at 13,000× *g* at 4 °C. The supernatant was transferred to a glass vial and analyzed by UHPLC-MS. A calibration curve was prepared in the same manner, by spiking known amounts of the synthetic standards XN and 8PN into serum prior to extraction. 

UHPLC-MS was performed at the Proteomics and Metabolomics Core Facility at Colorado State University on a Waters Acquity M-class UPLC equipped with a trap valve manager coupled to a Waters Xevo TQ-S triple quadrupole mass spectrometer. Chromatographic separations were carried out on a Waters Atlantis dC18 stationary phase (300 µM × 150 mM, 3 µM). Mobile phases were acetonitrile with 0.1% formic acid (B) and water with 2 mM ammonium acetate (A). The analytical gradient was as follows: time = 0 min, 45% B; time = 2.5 min, 70% B; time = 5.5 min, 70% B; time = 6 min, 100% B; time = 7 min, 100% B; time = 7.5 min, 45% B; time = 12 min, 45% B. Trapping was performed using a Waters Symmetry C8 stationary phase (300 µM × 50 mM, 5 µM). Loading time was 2 min at 25% B. Flow rate was 15 µL for both trapping and analytical separation. Injection volume was 2 µL. Samples were held at 5 °C in the autosampler, and the analytical column was operated at room temperature, near 21 °C.

The mass spectrometer was operated in selected reaction monitoring (SRM) mode, where a parent ion is selected by the first quadrupole, fragmented in the collision cell, then a fragment ion selected by the third quadrupole. Product ions, collision energies, and cone voltages were optimized for each analyte by direct injection of individual synthetic standards. A quantitative and confirmatory transition was developed for each analyte (Table 3). Interchannel delay was set to 3 ms. The instrument was operated in negative ionization mode and the capillary voltage was set to 2.1 kV. Source temperature was 150 °C and desolvation temperature 200 °C. Desolvation gas flow was 800 L/h, cone gas flow was 150 L/h, nebulizer gas flow was 7 Bar, and collision gas flow was 0.2 mL/min. Argon was used as the collision gas, otherwise, nitrogen was used.

### 2.5. Bile Acid Determination

To establish whether the various treatments resulted in functional changes to the microbiota with regard to bile acid metabolism, we measured a panel of twenty-one primary and secondary bile acids. Fecal samples (25 mg) from week 10 of the study, were homogenized in 500 L of NH_4_OH, with 5 L internal standards glycodeoxycholic acid d-4, deoxycholic acid d-4, and taurocholic acid d-5. The mixture was vortexed and incubated at 60 °C for 1 h, followed by sonication for 30 min. One mL of HPLC grade water was added and incubated at ‒80 °C overnight. Samples were then centrifuged at 4 °C at 10,000 rcf for 30 min. The clear supernatant was transferred for UHPLC-MS analysis. Analysis was performed at the Colorado State University Proteomics and Metabolomics Core Facility on a Waters (Millford, MA, USA) Acquity UHPLC coupled to a Waters Xevo TQ-S triple quadrupole mass spectrometer, as described previously [29]. Chromatographic separations were carried out on a Waters HSS T3 stationary phase (1 × 100 mm, 1.8 µM). The mobile phases were methanol (M) and water with 0.1% formic acid, and 2 mM ammonium hydroxide (A). The samples were held at 4 °C and column at 70 °C. The analytical gradient was carried out as follows: at 0 min, 0.1% M; time 0.5 min, 0.1% M; time 2 min, 30% M; time 15 min, 97% M; time 16 min, 97% M; time 16.5 min, 0.1% M; time 21 min, 0.1% M. Flow rate was 210 µL/min and injection volume was 2 µL. The mass spectrometer was operated in selected reaction monitoring (SRM) mode. Inter-channel delay was set to 3 ms and the MS was operated in both negative and positive ionization modes with a capillary voltage of 2.1 and 3.2 kV. The source temperature was 150 °C and the desolvation temperature was 500 °C, with a gas flow of 1000 L/h, cone gas flow of 150 L/h, and collision gas flow of 0.2 mL/min. Nebuliser pressure was 7 Bar and argon was used as the collision gas. Waters TargetLynx software was used for peak integration.

### 2.6. Short Chain Fatty Acid Determination

To determine whether the fermentative capacity of the gut microbiota was influenced by surgical intervention or supplement treatment, we measured fecal short chain fatty acids (SCFA), which are major end products of fiber fermentation. Fecal samples collected one week prior to study termination (11 weeks) were extracted for SCFA by homogenizing approximately 25 mg of frozen feces with acidified water (pH 2.5; adjusted with 12 M HCl) containing 10 mM ethylbutyric acid as an internal standard. Samples were vortexed and then sonicated for 60 min. After sonication, they were centrifuged at 10,000 rpm at RT for 15min. The supernatant was transferred to glass vials and stored at ‒80 °C, prior to GC-FID analysis. Sample extracts were analyzed on an Agilent 6890 Series Gas Chromatograph equipped with flame ionization detection (GC-FID; Agilent Inc., Santa Clara, CA, USA). The injection rate was 10:1 split ratio, inlet temperature was 22 °C and translet line temperature was held at 230 °C. Separation was achieved on a 30 m TG-WAX-A column (Thermo Scientific, 0.25 mm ID, 0.25 um film thickness) at 100 °C for 1min and ramp rate of 8 °C per minute to 180 °C, held at 180 °C for 1 min, ramped to 200 °C at 20 C/min and held for 5 min. Helium carrier flow was maintained at 1.2 mL per minute. SCFA were quantified using 5-point standard curves of commercially purchased standards (Sigma, St. Louis, MO, USA) and normalized to an internal standard signal.

### 2.7. Intestinal Permeability and Inflammation

One week prior to the termination of the study (11 weeks), intestinal permeability was assessed in vivo, as described previously [30]. In summary, mice were fasted for 6 h and orally gavaged with 40 kD FITC-Dextran (Sigma, St. Louis, MO, USA) dissolved in water at 400 mg/kg body weight. At 1 and 4 h time points, approximately 200 L of tail vein blood was collected and incubated in the dark at room temperature for 30 min. Samples were centrifuged at 5000× *g* at 4 °C for 10 min. Serum was removed and diluted with equal parts PBS. Fluorescence was read at 485 _EX_/535 _EM_ and concentration was calculated based on standard curve of serially diluted untreated serum spiked with FITC-dextran.

Intestinal alkaline phosphatase (IAP), which is an enzyme that is used to detoxify bacterial lipopolysaccharides (LPS), was measured in ileum tissue that was homogenized in a bullet blender and diluted in Bio-Plex Cell Lysis Kit buffer (BioRad, Hercules, CA, USA) by colorimetric assay against a standard curve, using SensoLyte pNPP Alkaline Phosphatase Assay Kit (AnaSpec Inc, Fremont, CA, USA) according to manufacturer’s instructions. LPS-binding protein (LBP) and soluble CD14 (sCD14), which are often used as proxy biomarkers for plasma LPS, were measured in homogenized and diluted ileum tissue by an ELISA assay, using Boster CD14 PicoKine and LBP PicoKine kits (Bosterbio, Pleasanton, CA, USA). Pro- and anti-inflammatory cytokines and chemokines IL-6, IL-10, IL-1β, MCP-1, MIP-1α, MIP-1β, IFNγ were measured in the proximal colon, which was homogenized and diluted, similar to the ileum tissue described above. Levels were measured by bead-based multiplex assay with Milliplex MAP Mouse Cytokine/Chemokine Magnetic Bead Panel kit (Millipore Sigma, Burlington, MA, USA) according to manufacturer’s instructions.

### 2.8. DNA Extraction and 16S Sequencing

Cecal contents from all study animals were collected with sterile cotton swabs at termination, flash frozen in liquid nitrogen, and stored at ‒80 °C until analysis. Whole genomic DNA was extracted using MoBio Powersoil DNA extraction kit (MoBio, Carlsbad, CA, USA) per manufacturer’s instructions. Extracted DNA was sent to Research Testing Laboratories (Lubbock, TX, USA) for library preparation by amplification of the V3-V4 ribosomal rRNA gene variable regions and sample indexing. Paired-end sequences were generated on an Illumina MiSeq platform (San Diego, CA, USA). Raw fastq data was processed using the DADA2 pipeline [31] using the software myPhyloDB version 2.0 [32] and sequence reads were normalized by rarefaction to 5000 reads per sample. 

### 2.9. Statistical Analysis

All statistical analyses were done using GraphPad Prism version 8.0.2 (GraphPad Software, La Jolla, CA, USA). Multiple comparisons among treatment groups are reported as standard error of the mean, using one-way ANOVA with Tukey post hoc comparisons, with statistical significance set at *p* < 0.05. Outliers were identified using the Rout method with Q = 1.0%. Phylogenetic Edge R differential abundance analysis was created in R, as a general linear model with treatment as main effect. 

## 3. Results

### 3.1. Weight Gain, Adiposity and Liver Triglycerides

**Figure 2:**
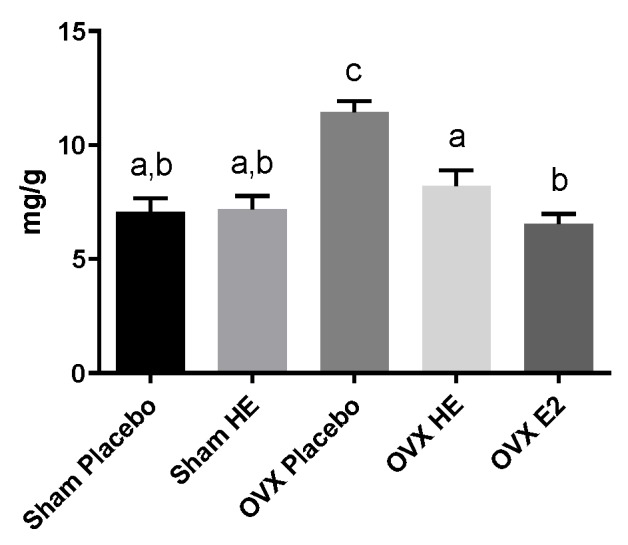
Liver triglycerides in milligrams per gram tissue. Bars denoted with unique letters have significant differences at *p* < 0.05.

Compared to Sham, OVX resulted in weight gain, although terminal body weight differences did not reach significance (Table 4). However, the OVX Placebo group had significantly higher visceral adipose tissue (VAT) compared to SHAM Placebo. The OVX E2 group was protected from this VAT increase, and the OVX HE also showed some protection, although they were trending towards a significant increase in VAT. Subcutaneous adipose tissue (SAT) and brown adipose tissue (BAT) were not significantly different. Likewise, there were no significant differences in cecal weights. Relative to SHAM Placebo, uterine weight was significantly decreased in OVX Placebo, OVX HE and OVX E2 groups (Table 4). The OVX Placebo group had significantly higher levels of liver triglycerides (TG) than all other groups (*p* < 0.001, Figure 2), whereas liver TG in OVX HE, SHAM HE and OVX E2 were not significantly different from SHAM Placebo. Liver tissue was assessed for gene expression for several lipid transport and metabolism genes, including ATP-binding cassette subfamily G (ABCG5 and ABCG8), steroid regulatory binding protein 1c (SREBP1c), fatty acid synthase (FASn), hormone sensitive lipase (HSL), and acetyl-CoA carboxylase (AcCoA). The OVX HE group had lower FASn and AcCoA carboxylase expression, though differences amongst groups did not reach significance (data not shown). 

### 3.2. Pharmacokinetics of Hop Prenylvlavonoids in Serum

**Figure 3:**
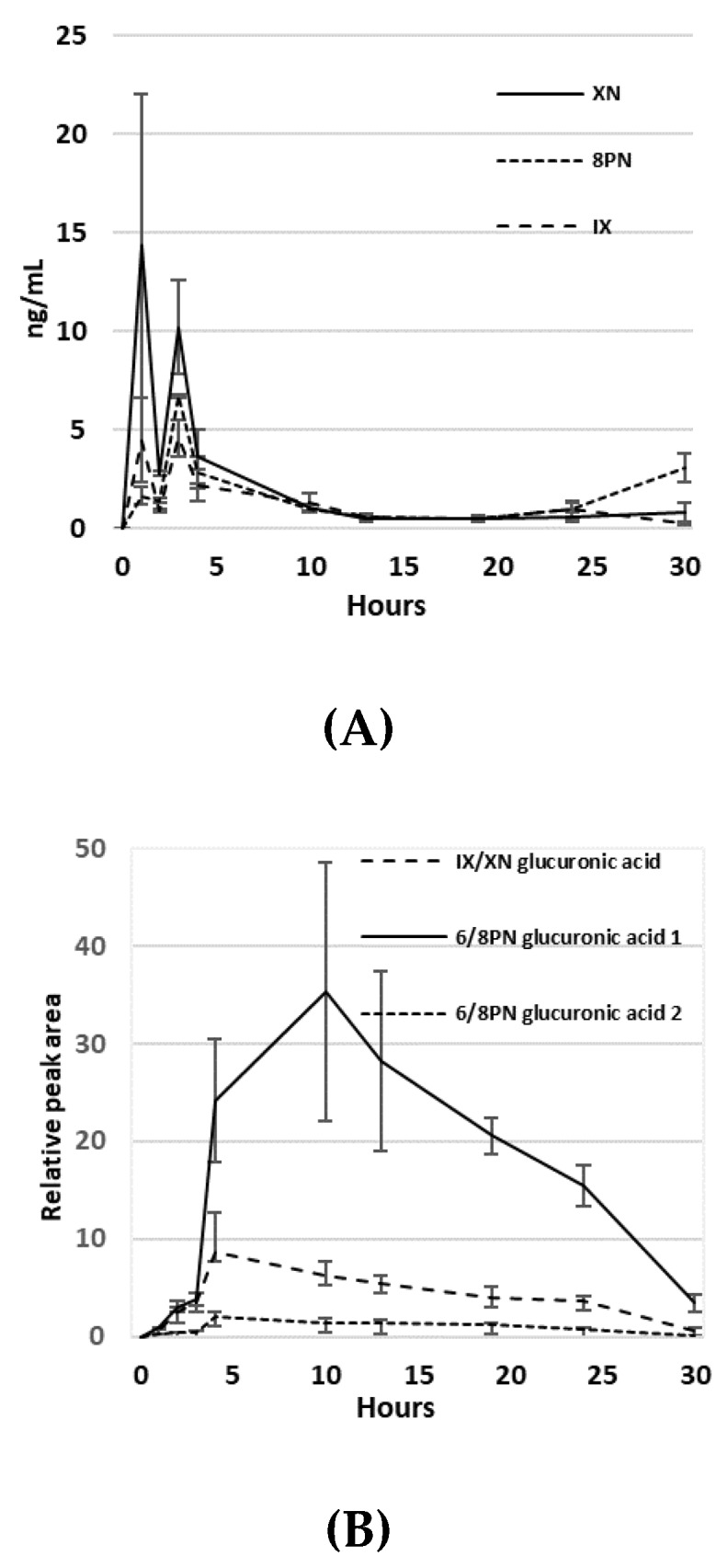
(**A**) Pharmacokinetics of XN, IX and 8PN in serum after oral administration of hop extract in ng/mL; (**B**) Pharmacokinetics of glucuronidated compounds of XN, IX, 8PN and 6PN. Values are relative peak area.

Circulating blood levels of HE were quantified by LC-MS. The pharmacokinetics of XN, IX and 8PN were quantified against known standards, while values of their glucuronidated species are based on relative peak area. Serum in non-study mice (*n* = 5) was measured after oral HE administration over a period of 30 h. XN had a detected T*_max_* of 1 h, with a detected C*_max_* = 14.33 ng/mL. IX and 8PN both had a T*_max_* of 3 h, with a detected C*_max_* = 4.58 ng/mL and 6.73 ng/mL, respectively (Figure 3A). Three glucuronidated species were also identified (Figure 3B). Measurements are reported as relative peak area, since known standards were not available to calibrate signal intensity. The glucuronidated compounds had a T*_max_* = 4 h for IX/XN-glucuronic acid and 6PN/8PN-glucuronic acid, while another chemically distinct form of 6PN/8PN-glucuronic acid detected had a T*_max_* = 10 h.

### 3.3. Bile Acid and Short Chain Fatty Acid Quantification

There were no statistically significant differences in fecal bile acids amongst groups (*n* = 5/group). One mouse in the OVX Placebo was a consistent outlier in levels of cholic, deoxycholic, ursodeoxycholic, chenodeoxycholic and glycocholic acids, and was not included in the analysis. Cholic acid, deoxycholic acid, ursodeoxycholic acid and chenodeoxycholic acid were highest in the OVX HE group and below detection in OVX E2 group (Figure S1). There were also no significant differences in fecal short chain fatty acid (SCFA) levels amongst groups (Figure S2). Acetate was detected in the majority of the samples, however, many samples were below the level of detection for butyrate and proprionate. This may be because butyrate is absorbed by the colonic epithelial cells as a fuel source, and proprionate is shuttled to the liver, leaving feces relatively depleted [33]. 

### 3.4. Intestinal Function

**Figure 4:**
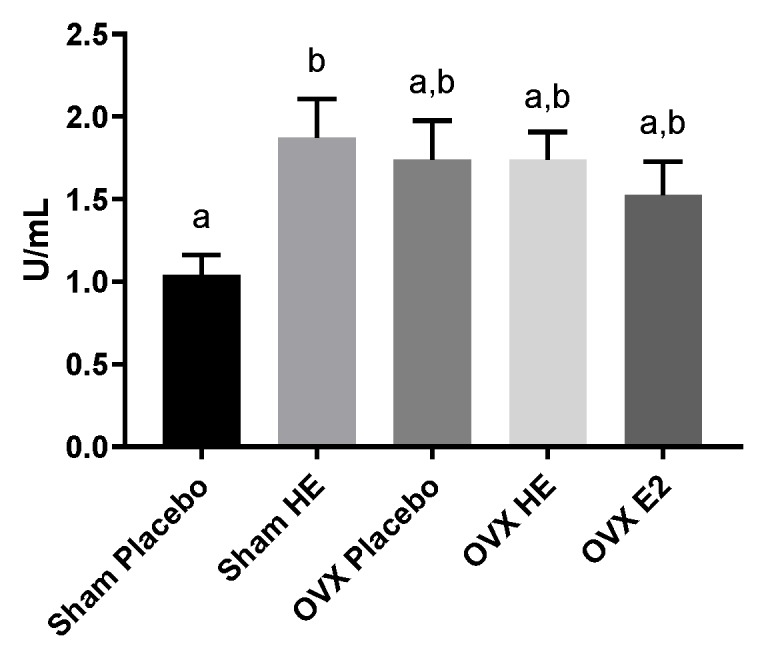
Intestinal alkaline phosphatase levels in ileum. Bars denoted with unique letters have significant differences at *p* < 0.05.

Measurement of intestinal barrier function using FITC-dextran revealed no differences between groups, with most samples having fluorescent measurements consistent with background readings (data not shown). LPS-binding protein (LBP) and soluble CD14 (sCD14) dimerize to bind to circulating LPS, and increased levels in the plasma are indicative of higher circulating LPS levels and endotoxemia [34], making these viable proxy measures for LPS. Post-mortem measurement of plasma LBP and sCD14 was not significantly different among groups (Figure S3). The levels of intestinal alkaline phosphatase (IAP), an enzyme that plays a role in the neutralization of luminal endotoxin, were similar amongst the three OVX treatment groups and were not significantly different from Sham Placebo. However, IAP was significantly higher in the Sham HE compared to the Sham Placebo group (*p* = 0.04; Figure 4). Intestinal inflammation was assessed by examining colonic cytokines and chemokines. IL-6 and IL-10 levels were highest in the OVX HE group, although levels of IL-6, IL-10, IL-1β and MCP-1 did not reach statistical significance (Figure S4). Levels of IFN-γ, MIP-1α, MIP-1β were below detection in most samples (data not shown).

### 3.5. Microbiota Analyses

**Figure 5:**
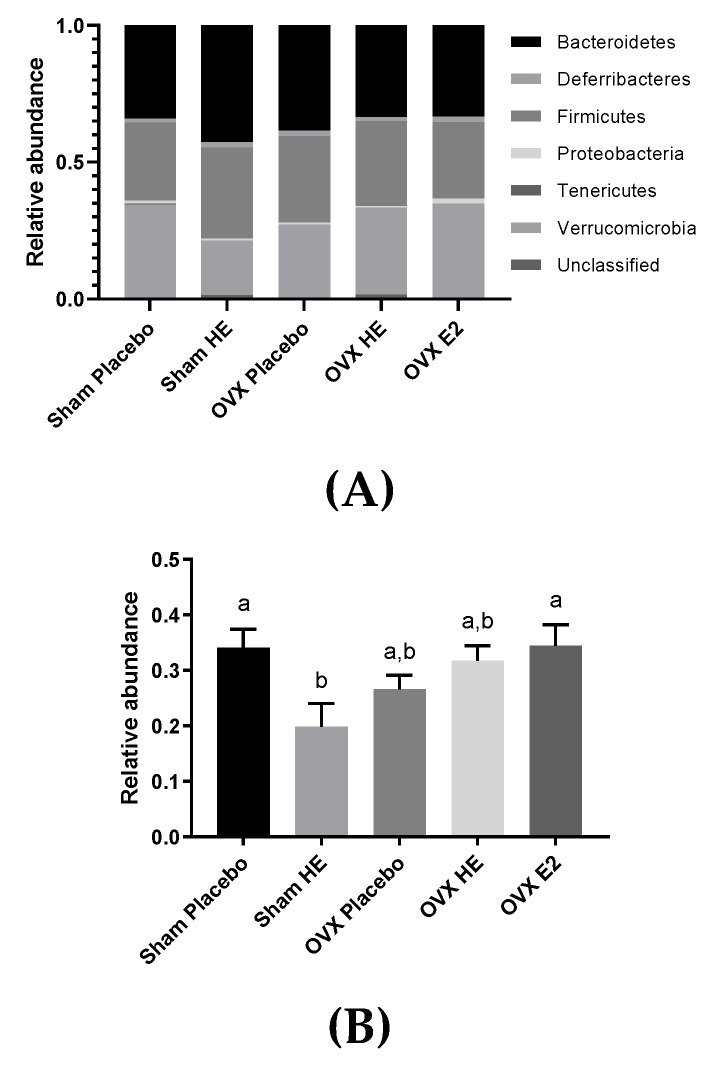
(**A**) Relative abundance of bacterial phyla in the cecum. (**B**) Microbial relative abundance of *Akkermansia muciniphila* in the cecum. Bars denoted with unique letters have significant differences at *p* < 0.05.

There were no alterations in the gut microbial community structure (alpha- and beta-diversity), as a result of OVX and E2 or HE supplementation, in cecal contents collected upon study termination. Cecum weights were similar amongst all groups, suggesting that bacterial abundances were also similar, although this was not confirmed by qPCR. There was one notable difference at the phyla level: Verrucomicrobia was lower in Sham HE compared to the Placebo and OVX E2 groups (Figure 5A). Verrucomicrobia contains only one identified species, *Akkermansia muciniphila* (Figure 5B), and the relative abundance of this species was also lower in Sham HE group compared to the Sham Placebo (*p* = 0.03) and OVX E2 (*p* = 0.04).

## 4. Discussion

The onset of menopause is associated with increased risk of several chronic diseases of the cardiovascular system, bone and insulin-sensitive tissues. The microbial composition of the gut has been similarly identified as a modulator of these conditions. Given the interest in alternative treatments for menopause-associated side effects, and the role of the gut microbiota in the metabolism estrogen and phytoestrogenic compounds, such as those in hops, we sought to investigate these interactions in an ovariectomized mice. Menopause is often marked by an increase in body weight, without change in diet or exercise habits. Although it did not reach statistical significance relative to the SHAM group, there was a trend toward overall body weight increase with OVX, as previously reported [35,36,37]. However, there was a significant increase in visceral adipose tissue (VAT) with OVX. Supplementation with E2 and, to a lesser extent, HE, protected against accumulation of VAT in these animals. Other flavonoids have been shown to mitigate increases in visceral adipose tissue, such as green tea extracts (*Camellia sinensis*) [38,39], red wine grapes [40], and licorice root [41]. Many of these studies employed diets high in saturated fat, therefore it is interesting that HE exerted protective effects in the present study, where mice were on a low-fat diet. VAT is pro-inflammatory and is associated with increased risk of several cardiometabolic diseases and cancers [42,43,44], so preventing or reducing its accumulation is beneficial. It is not surprising that E2 treatment decreased adiposity, since estrogen is involved in leptin signaling and other regulators of metabolic homeostasis [45,46]. We hypothesized that HE would have similar protective effects due to the estrogenic activity of 8PN. The reduced effect may be attributed to the incomplete conversion of IX to 8PN. Our sequencing analysis did not detect bacterial species (*Eubacterium limosum* and *E. ramulus*) previously reported as converters of IX to 8PN, and our own pharmacokinetic data suggest a high degree of variability between animals with regard to the bioavailability of the HE compounds.

We were able to detect the major HE flavonoids, as well as several glycosylation products, in mouse serum. The bi-modal peaks in XN and IX suggest these compounds are absorbed in both the small intestine and colon. The levels of XN were highest at 1 h after HE ingestion, while IX and 8PN were higher at 3 h post-consumption. This spontaneous conversion of XN to IX, followed by enterohepatic circulation and conversion of IX to 8PN by liver microsome CYP1A2 [47] or microbial metabolism by colonic bacteria [24,25,48]. These results are consistent with van Breemen et al., who measured the pharmacokinetics of oral HE in women [23], and provide evidence that mice in the current study were exposed to flavonoid levels capable of exerting physiological effects. Hop flavonoids, especially XN, have been shown to be protective against high-fat-diet-induced accumulation of liver triglycerides, modulated via regulation of genes involved in fatty acid and cholesterol metabolism [49,50,51]. We did observe a reduction in liver triglycerides in HE-treated animals, however, we could not confirm that these were due to altered expression of genes involved in lipid metabolism. It is possible the reduction in liver triglycerides was mediated through other pathways. Naringenin, a flavonoid structurally similar to 8PN, was found to activate both PPARα and PPARγ while inhibiting LXRα in rat hepatocytes, regulating downstream fatty acid oxidation genes [49].

We have previously demonstrated in a rat model that OVX was accompanied by alterations in the gut bacteria [6]. In this study, we hypothesized that OVX would alter the mouse gut microbiota and that HE and E2 would prevent or minimize these effects. However, we saw no significant differences in alpha or beta diversity across treatment groups and the community composition and size, inferred from a lack of differences in cecal weights, was similar, even at finer taxonomic scales. The lack of differences, particularly between OVX and SHAM groups was unexpected, particularly as OVX was accompanied by increased visceral adiposity and several previous studies have reported OVX-associated microbial shifts [52]. However, there are multiple environmental factors that can influence the microbiota, such as diet, age, and time post-OVX, that may account for differences between our study and previous reports. Additionally, differences in sequencing depth and sequence-processing pipelines could account for this discrepancy. As retired breeders, the mice in the current study were also multi-parous, whereas most OVX studies are in virgin animals. While we are not aware of any studies investigating the difference in gut microbiota between nulli- and multiparous subjects (human or animal), there is some evidence that the vaginal microbiome is associated with parity [53]. Finally, there are also reports that external trauma, such as burns, sepsis, and surgery, cause changes to the gut microbiota and intestinal environment [54], and so it is possible that there were surgery-associated inflammatory impacts to the intestinal environment in both groups of animals. However, we did not collect fecal samples for sequencing prior to the surgical intervention, making this a limitation of the current study.

The only notable microbiota difference we detected was a significant reduction in *Akkermansia muciniphila* in SHAM HE compared to SHAM placebo and OVX E2 (Figure 5B). The growth of this species may have been influenced by interactions between endogenous estrogen and HE supplementation, as this reduction was not observed in OVX HE animals. This idea is supported by Chen et al., who observed a decrease in *A. muciniphila* in OVX mice supplemented with combined conjugated estrogens plus Bazedoxifene (a selective estrogen receptor modulator), along with a decrease in deconjugation enzymes [55]. *Akkermansia muciniphila* utilizes intestinal mucin as its sole energy source [56] and the degradation of mucins stimulates the regeneration of new mucin by epithelial cells, and releases mucin metabolites that are utilized by other bacteria in the lumen. Furthermore, higher levels of *A. muciniphila* are associated with a lower body weight in humans and mice [57,58,59], and *A. muciniphila* is positively associated with lower levels of blood glucose, LDL cholesterol and triglycerides [60]. Moreover, probiotic feeding of live, but not heat-killed, *A. muciniphila* reversed high-fat-diet-induced metabolic disorders and increased endocannabonioid levels in mice, suggesting an important role in the regulation of host metabolism, inflammation and gut barrier function [61]. Thus, further investigation of the effects of HE on these bacteria may be warranted. 

In addition to measuring changes in the gut microbiota, we also measured changes in microbial function (microbial metabolites) and intestinal barrier integrity and inflammation. Gut dysbiosis is often characterized by a detrimental alteration to the gut microbiota and is associated with increased markers of inflammation and reduced barrier function [62]. We did not see major differences in the microbial community structure, diversity or richness, between SHAM and OVX animals, and therefore, it is not surprising that we did not see differences in barrier function or markers of inflammation. Flavonoids interact with exogenous antioxidant and anti-inflammatory pathways [63]. It is interesting that we saw an increase in IAP with HE only within the Sham group. This is consistent with the reduction in *A. muciniphila* in this group and the increase in IAP, which is known to mitigate intestinal inflammation and bacterial translocation, and may be a compensatory response to the reduced barrier protection provided by *A. muciniphila*.

## 5. Conclusions

The menopause is associated with a marked increase in overall body weight, visceral adiposity, and triglyceride accumulation in the liver and circulating blood [64,65,66], known risk factors for cardiometabolic disease and cancer in humans [67,68]. Accumulating evidence in animal models suggests that estrogen-mediated impacts on gut bacteria may contribute to these physiologic changes. However, there were no significant OVX-related effects on the gut microbiota in our study, suggesting that short-term estrogen loss per se is not detrimental to the gut microbiota, but may be dependent on other physiological and environmental parameters, such as parity and diet. In humans, the menopausal transition is associated with gradual and sustained estrogen reductions as well as changes in other gonadal hormones and associated metabolic adaptations. Therefore, studies using OVX mice as a model for menopause in humans must be interpreted with caution.

Independent of the gut microbiota, HE and E2 provided protection against menopause-associated visceral adiposity and liver triglyceride accumulation. Flavonoids in HE, most notably XN, are known modulators of lipid metabolism [69,70,71] and inflammatory cytokines [72,73,74]. Since menopause-associated adiposity and liver triglyceride accumulation poses a significant risk for the development of cardiometabolic disease, HE could be an appropriate treatment for women undergoing menopause. It has been demonstrated in human models that HE is effective at relieving uncomfortable side effects of the menopause, including hot flashes, insomnia and mood swings [21,75]. However, there has not been a study in menopausal women on the effectiveness of HE in reducing or preventing menopause-associated adiposity. Future studies in human and murine models are needed to delineate the effects of HE on menopausal side-effects, as well as obesity-related disease risk.

## Figures and Tables

**Table 1:** Average daily caloric and macronutrient intake.

	Daily Intake (g)	Total Calories	% Calories Fat	% Calories Protein	% Calories Carbohydrate
Diet	2.99	10.75	10%	14%	76%
Cookie	0.20	1.04	49%	5%	48%
Oil	0.03	0.27	100%	0%	0%
Total:	3.22	12.06	15.6%	12.7%	71.9%

**Table 2:** Primer Sequences used for gene expression.

Target Gene	Sequence
**ABCG5**	For: 5′-CAGGGACCGAATTGTGATTG-3′
	Rev: 5′-GAACACCAACTCTCCGTAAG-3′
**ABCG8**	For: 5′-CTGGAATCCTGAGAGGATAG-3′
	Rev: 5′-TAGGTCGCCCTTTGTATTGG-3′
**FASn**	For: 5′- AGACTACAGACGACAGCAACC-3′
	Rev: 5’-CTCTCAGACAGGCACTCAGC-3’
**SREBP1c**	For: 5′-TGGTGGGCACTGAAGCAAAG-3′
	Rev: 5′-CACTTCGTAGGGTCAGGTTCTC-3′
**AcCoA carboxylase**	For: 5′-CTTCGCCATAACCAAGTAGAG-3′
	Rev: 5′-GTTTCCGAGAGGATGAGTTTC-3′
**Hormone sensitive lipase**	For: 5′-CAGTCAATGGAGACACTTGG-3′
	Rev: 5′-GGGTCTCACTTCATCTTTGG-3′

**Table 3:** UHPLC-MS quantitative and confirmatory transition for each analyte measured in serum. CV; cone voltage, CE; collision energy.

Molecule	Parent *m*/*z*	Fragment *m*/*z*	CV	CE
Xanthohumol	353.3	119	50	26
Xanthohumol	353.3	233.2	50	18
8-prenylnaringenin	339.3	219.2	60	22
8-prenylnaringenin	339.3	119	60	30
Naringenin	271.2	151.1	50	18
Naringenin	271.2	119	50	26
2,4-dihydroxychalcone	239.2	135.1	60	22
2,4-dihydroxychalcone	239.2	197.1	60	20
Xanthohumol-glucuronic acid	529.3	353.3	50	25
8-prenylnaringenin-glucuronic acid	515.3	339.3	50	25

**Table 4:** Tissue weights at study termination.

	Sham Placebo	Sham HE	*p*-Value	OVX Placebo	*p*-Value	OVX HE	*p*-Value	OVX E2	*p*-Value
BW	31.0	30.1	0.96	34.3	0.09	33.9	0.16	31.0	0.99
Cecum	296.7	317.6	0.99	243.9	0.91	274.7	0.99	297.7	0.99
Uterus	107.7	89.5	0.32	28.0	<0.001 *	31.2	<0.001 *	78.9	0.023 *
SAT	1.24	1.17	0.99	1.67	0.21	1.54	0.57	1.16	0.99
BAT	0.11	0.12	0.99	0.14	0.78	0.15	0.55	0.13	0.93
VAT	2.259	2.288	0.99	3.68	<0.001 *	3.27	0.063	2.02	0.97
VAT/Total AT	0.627	0.638	0.98	0.671	0.09	0.655	0.48	0.608	0.89

One-way ANOVA using Tukey post-hoc correction for multiple comparisons to Sham Placebo group. All weights are in milligrams except body weight, which is in grams. Asterisks (*) indicate significant difference to Sham Placebo at *p* < 0.05. BW; body weight, SAT; subcutaneous adipose tissue, BAT; brown adipose tissue, VAT; visceral adipose tissue, AT; adipose tissue.

## Supplementary Materials

The following are available online at https://www.mdpi.com/2072-6643/11/12/3004/s1, Figure S1: Levels of bile acids in fecal samples; Figure S2. Levels of short chain fatty acids (SCFA) in fecal samples; Figure S3: Blood measures of endotoxin binding proteins LBP and soluble CD14; Figure S4: Cytokine and chemokine levels in proximal colon.
