# Supplementary material for: The Effect of Hops (Humulus lupulus L.) Extract Supplementation on Weight Gain, Adiposity and Intestinal Function in Ovariectomized Mice
Source: Nutrients. 2019 Dec 7;11(12):3004. doi: 10.3390/nu11123004 (PMC6950254; doi:10.3390/nu11123004)
Supplement: Supplementary file 1 [file nutrients-11-03004-s001.pdf]

## Supplementary Data

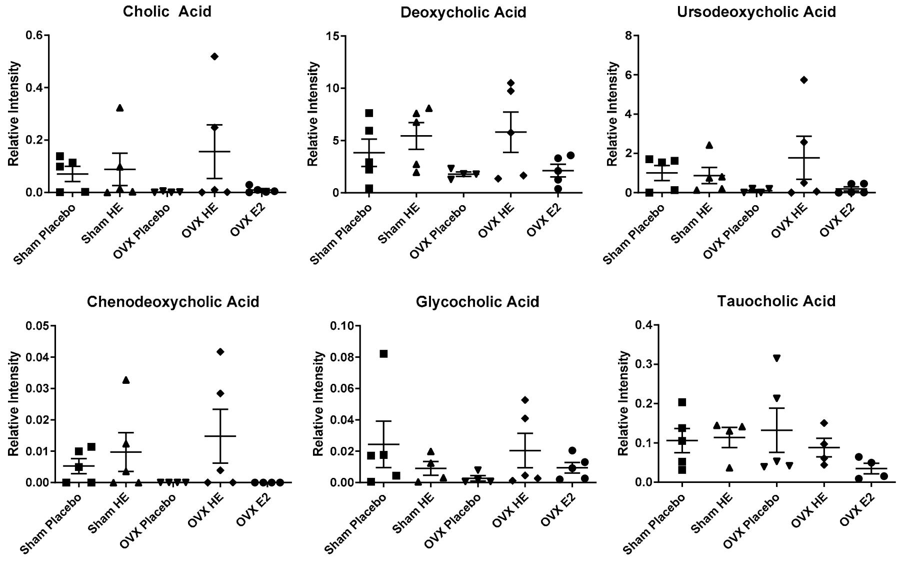


**Figure S1.** Levels of bile acids in fecal samples. .

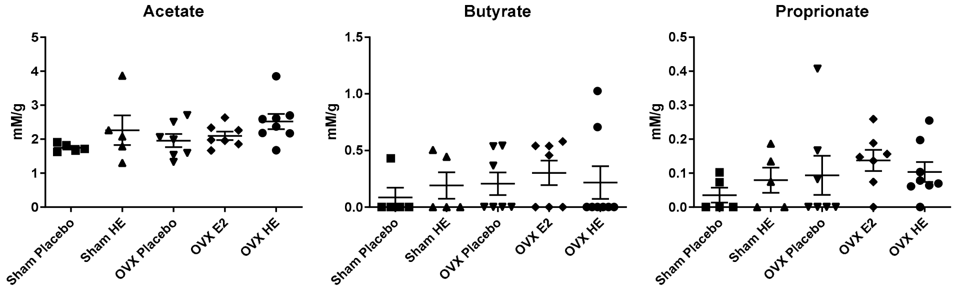


**Figure S2.** Levels of short chain fatty acids (SCFA) in fecal samples.

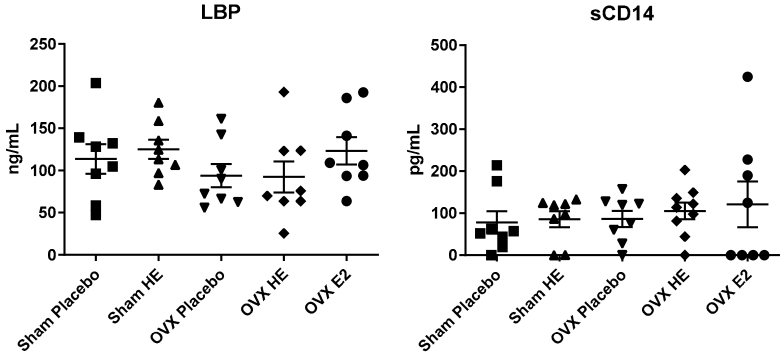


**Figure S3.** Blood measures of endotoxin binding proteins LBP and soluble CD14.

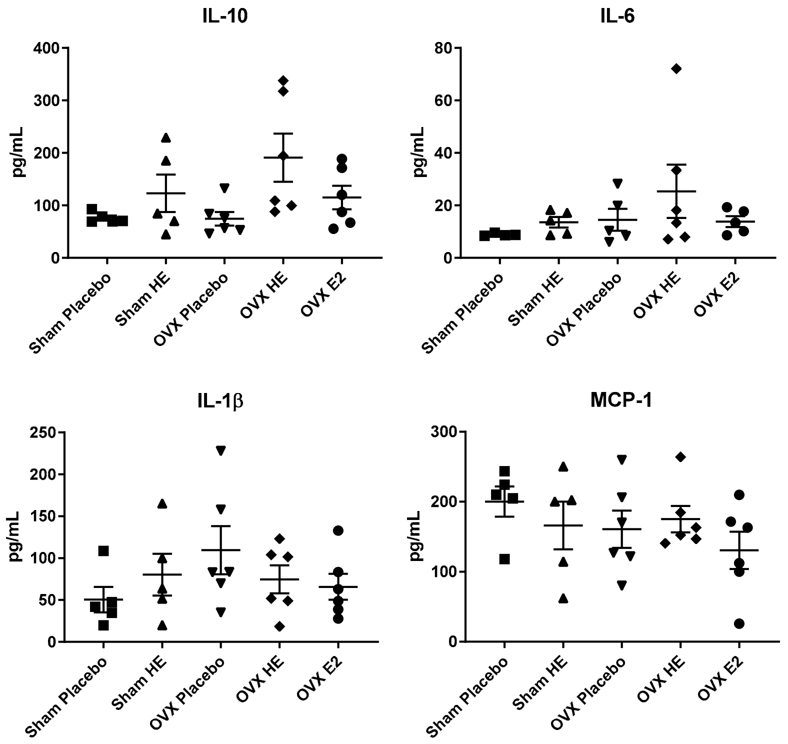


**Figure S4.** Cytokine and chemokine levels in proximal colon.
